# Twenty minutes of Corsi block tapping task training does not improve mental rotation in adults with stroke

**DOI:** 10.3389/fneur.2025.1601454

**Published:** 2025-10-16

**Authors:** Sarah A. Kettlety, Giuliet L. Kibler, Andrew Hooyman, Christina K. Holl, Sydney Y. Schaefer, Kristan A. Leech

**Affiliations:** ^1^Division of Biokinesiology and Physical Therapy, University of Southern California, Los Angeles, CA, United States; ^2^Department of Physical Therapy, Crean College of Health and Behavioral Science, Chapman University, Irvine, CA, United States; ^3^School of Biological and Health Systems Engineering, Arizona State University, Tempe, AZ, United States; ^4^Neuroscience Graduate Program, University of Southern California, Los Angeles, CA, United States

**Keywords:** stroke, cognition, visuospatial function, cognitive training, mental rotation

## Abstract

**Background:**

Visuospatial function is commonly impaired post-stroke and is associated with motor learning and recovery. A single, twenty-minute Corsi Block Tapping Task (CBTT) training session improved visuospatial function in young neurotypical adults; however, it is unclear whether this training would improve visuospatial function in adults with stroke.

**Objective:**

To understand if a single, twenty-minute CBTT training session improved visuospatial function in adults with stroke compared to a no-training control group of adults with stroke.

**Methods:**

Participants post-stroke were assigned to one of two groups. The training group completed twenty minutes of computerized CBTT training. The control group completed a survey and watched a video for twenty minutes. Both groups completed a mental rotation task to assess visuospatial function pre- and post-training. To understand if training impacted mental rotation reaction time, we fit a robust mixed effects model with fixed effects for time, group, and time by group interaction. We also investigated whether lesion side impacted CBTT performance using a robust mixed effects model with fixed effects for log(time), lesion side, and log(time) by lesion side interaction.

**Results:**

Nineteen participants post-stroke were included. Neither the control nor training group improved mental rotation reaction time (time *p* = 0.61, group *p* = 0.65; interaction *p* = 0.52). We also found no effect of lesion side on CBTT performance [log(time) *p* = 0.001, lesion side *p* = 0.49, interaction *p* = 0.89].

**Discussion:**

Twenty minutes of CBTT training did not improve post-stroke mental rotation. Longer training bouts or a different type of visuospatial training may be necessary to improve visuospatial function in adults with stroke.

## 1 Introduction

Visuospatial function is commonly impaired in adults with stroke (1, 2) and is associated with lower quality of life (3–5), reduced participation (6), and difficulty completing activities of daily living (5, 6). Visuospatial function broadly reflects someone’s ability to perceive the spatial properties of a two- or three-dimensional figure or object (7). However, different components of visuospatial function can be measured separately including visuospatial/constructional skills and visuospatial working memory. Visuospatial/constructional skills represent someone’s ability to perceive a visual image, break it down into parts, and reconstruct the image (8). Visuospatial/constructional skills are commonly measured using the Repeatable Battery for the Assessment of Neuropsychological Status (RBANS) (9) and the Rey-Osterrieth Complex Figure Test (10). Visuospatial working memory represents the ability to store and manipulate visual information (11). It is commonly measured using the Corsi Block Tapping Task (CBTT) (12) and the Spatial Addition from the Wechsler Memory Scale-IV (13).

Evidence suggests that both visuospatial/constructional skills and visuospatial working memory may be related to motor learning in neurotypical adults (14–21). Visuospatial/constructional skills are related to one-week retention of an upper extremity task in neurotypical older adults (15). Visuospatial working memory is associated with skill acquisition (17), one-week retention (17), and one-month retention (14, 19) of a functional upper extremity task in neurotypical older adults. Visuospatial working memory is also related to sensorimotor adaptation and motor sequence learning (18).

Motor learning is the foundation of many post-stroke rehabilitation interventions (22). Outcomes from post-stroke rehabilitation studies are often variable between individuals (23, 24). Variability in post-stroke motor learning likely arises from multiple factors, one possibly being cognitive impairment (25). Specifically, there is some evidence that visuospatial function may impact motor learning in individuals post-stroke (14, 26). Visuospatial/constructional skills are related to performance on a gait biofeedback task in adults post-stroke (26) and visuospatial working memory predicts one-month retention of a functional upper extremity task in adults with stroke (14). Additionally, visuospatial/constructional skills have been linked to long-term functional rehabilitation outcomes post-stroke (27, 28). Combined, these results suggest a link between visuospatial function, motor learning, and functional rehabilitation outcomes; thus, improving visuospatial function with targeted interventions may have downstream effects on motor learning during rehabilitation after stroke.

One potential way to improve visuospatial function after stroke may be through computerized visuospatial training paradigms. Recent work demonstrated that a single, twenty-minute visuospatial training session (using a computerized version of the CBTT) is sufficient to improve mental rotation abilities in neurotypical young adults (29). This suggests that improving visuospatial function is possible with a short training bout. However, whether twenty minutes of visuospatial training is adequate to improve visuospatial function post-stroke is unclear.

Here, we aimed to understand if a single, twenty-minute CBTT training session improved mental rotation performance (measured using reaction time) in adults with stroke compared to a no-training control group of adults with stroke. Given that mental rotation reaction time improved in neurotypical young adults after twenty minutes of CBTT training (29), we hypothesized that a single, twenty-minute CBTT training session would improve mental rotation performance (i.e., reduce reaction time) compared to a no-training group of adults with stroke.

## 2 Materials and methods

### 2.1 Participants

Twenty-two individuals at least six months post-stroke were recruited. Participants were recruited from an established, IRB-approved database of people living with stroke who have an interest in participating in research, as well as through local outpatient therapy clinics by our network of clinical partners. Inclusion criteria for participation included age eighteen to eighty, paresis confined to one side, and no orthopedic or pain conditions in the hands. Exclusion criteria included damage to the pons, basal ganglia, or cerebellum, signs of cerebellar involvement or extrapyramidal symptoms, hemispatial neglect, and a Montreal Cognitive Assessment five-minute protocol score of less than nineteen (26, 30). Written informed consent was provided before participation. The University of Southern California Institutional Review Board approved the study procedures.

### 2.2 Assessment of cognitive status

Measures of immediate memory, visuospatial/constructional skills, language, attention, and delayed memory were assessed with the RBANS (9). The RBANS has been found to be an appropriate test to detect domain-specific cognitive impairment post-stroke (31). Data for all participants were age-normalized using the RBANS scoring manual. For all domains, higher scores indicate better cognitive function. All RBANS were independently double-scored to identify and resolve any discrepancies in scoring.

### 2.3 Mental rotation task

**Figure 1:**
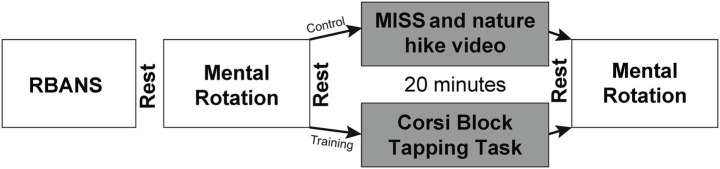
Experimental paradigm. RBANS, Repeatable Battery for the Assessment of Neuropsychological Status; MISS, Multidimensional Iowa Suggestibility Scale.

We used the computer-based Mental Rotation Task from the open-source Psychology Experiment Building Language (32) for our primary visuospatial function measure. The protocol for the mental rotation task is described in detail in previous work (29). Briefly, the mental rotation task presents participants with a pair of 2D asymmetrical objects rotated with respect to one another. The participants were asked to identify whether the two objects were identical as quickly as possible using the right and left arrows on the keyboard. The original protocol used the left and right shift keys, but the right and left arrows made the task unimanual and more attainable for participants post-stroke. Each participant completed 64 trials each session with four additional practice trials (none included in the analyses). The participant had 3,000 ms to provide an answer. Feedback (correct or incorrect) was presented 500 ms after each response. The trial was marked incorrect if the participant did not answer within 3,000 ms. The outcome measures were reaction time for correctly completed trials and number of correctly completed trials. After completing the initial mental rotation task, participants were randomly assigned to the training or control group. After twenty minutes, both groups repeated the mental rotation task (Figure 1).

### 2.4 Corsi block tapping task

The experimental group completed twenty minutes of visuospatial training using the CBTT from the Psychology Experiment Building Language (32). The protocol for the CBTT training is described in detail in previous work (29). Briefly, the CBTT is a visuospatial working memory task that presents nine square blocks (12) on the computer screen (32). During each trial, blocks sequentially lit up in yellow. The participant was asked to remember the sequence, then click on each square in the same order they were given. The task’s difficulty increased by increasing the sequence length when participants clicked the correct sequence twice in a row. Participants began their training with a sequence of three blocks and increased to a maximum of nine-block sequences based on performance. Once a nine-block sequence was reached, the sequence would remain nine blocks for the rest of the training. The primary outcome of this task was the best span (highest number of blocks correctly memorized) per trial.

### 2.5 Control condition

The control group completed a computerized version of the Short Suggestibility Scale, a subscale of the Multidimensional Iowa Suggestibility Scale (33). If the participants completed the questionnaire before the twenty-minute block was complete, a nature walk video was played on the computer for the remainder of the twenty minutes. The control paradigm engaged participants on the computer screen with minimal visuospatial demands. All participants in the control group were adults post-stroke.

### 2.6 Statistical analysis

All statistical analyses were performed in RStudio (R version 4.4.1) (34). To determine if CBTT training impacted mental rotation reaction time, we used a linear mixed effects model with fixed effects for time (pre- and post-), group (control and training), time by group interaction, and a random intercept for participant. We also included a fixed effect for sex to account for sex differences in mental rotation that have been previously reported (35, 36). We checked model assumptions using the performance package (37). The model included outliers (determined using the check_outliers function (37)); thus, we fit a robust linear mixed effects model to downweight the effect of these outliers (38). We also examined whether the number of correct mental rotation trials changed after CBTT training. We fit the same model described above, with the number of correct trials as the outcome.

To provide preliminary effect sizes to inform future work, we calculated Hedges’ g for change in reaction and change in number of correct trials between groups (39). To assess the overall within-subject changes in mental rotation reaction time, we also computed an effect size for paired samples (40).

Since the right hemisphere plays a large role in spatial memory (41–43), and previous work has demonstrated that lesion side impacted non-computerized CBTT performance (44), we also performed an exploratory analysis examining whether stroke lesion side impacted CBTT performance. We fit a linear mixed effects model with fixed effects for time (in minutes), lesion side, and a random slope for time and intercept for participant. Before fitting the model, we log-transformed time because participants experienced larger performance gains in early training and smaller gains later in training. To ensure the log transformation of time was appropriate, we compared a model with the logarithmic transformation of time and a linear model using Bayesian Information Criteria (BIC). We checked model assumptions using the performance package (37). The model included outliers; thus, we fit a robust linear mixed effects model to downweight the effect of these outliers (38).

## 3 Results

We included nineteen participants in the analysis. Ten participants were included in the control group (age: 51 ± 14, sex: 4 female, years since stroke: 6 ± 3, side affected: 5 left, RBANS total: 83 ± 11, RBANS visuospatial/constructional: 81 ± 15) and nine participants were in the training group (age: 58 ± 14, sex: 4 female, years since stroke: 8 ± 4, side affected: 4 left, RBANS total: 80 ± 11, RBANS visuospatial/constructional: 75 ± 11). There was no difference between groups in baseline cognitive function, measured by the RBANS total score (*β* = 2.6, *p* = 0.62). Of the 23 participants recruited, one participant was excluded from the training group due to hemispatial neglect, one was excluded from the control group due to technical difficulties, and one was excluded from the training group due to not improving past the first CBTT sequence. Participant demographics are included in Table 1.

**Table 1:** Participant demographics.

ID	Group	Age	Years since stroke	Side affected	RBANS total	RBANS IM	RBANS V/C	RBANS language	RBANS attention	RBANS DM
1	Control	31	10	Left	106	109	100	103	106	107
2	Training	45	6	Right	63	78	60	88	46	81
3	Training	62	15	Left	82	83	78	90	82	102
4	Training	61	5	Left	82	100	69	75	91	98
5	Control	38	3	Right	72	65	84	85	72	83
6	Control	45	9	Left	82	109	64	91	88	81
7	Control	33	4	Left	72	69	96	90	49	85
8	Control	67	8	Left	86	114	65	84	92	90
9	Training	58	2	Right	67	57	78	68	85	82
10	Control	50	3	Right	95	103	96	87	100	101
11	Training	75	6	Right	83	98	75	90	105	85
12	Training	49	11	Left	79	85	66	75	94	95
13	Control	52	3	Right	73	69	56	117	72	78
14	Training	64	0.75	Right	82	65	96	87	95	98
15	Training	74	0.67	Right	102	126	82	90	101	108
16	Control	65	10	Right	89	111	88	76	91	96
17	Control	62	6	Left	74	83	78	78	75	84
18	Control	64	2	Right	80	81	81	78	82	98
19	Training	32	8	Left	83	81	69	112	91	83
		54 (14)	6 (4)	9 L/10R	82 (11)	89 (19)	78 (13)	88 (12)	85 (16)	91 (9)

Abbreviations: RBANS, Repeatable Battery for the Assessment of Neuropsychological Status; IM, immediate memory; V/C, visuospatial/constructional; DM, delayed memory.

### 3.1 Twenty minutes of CBTT training did not improve mental rotation

**Figure 2:**
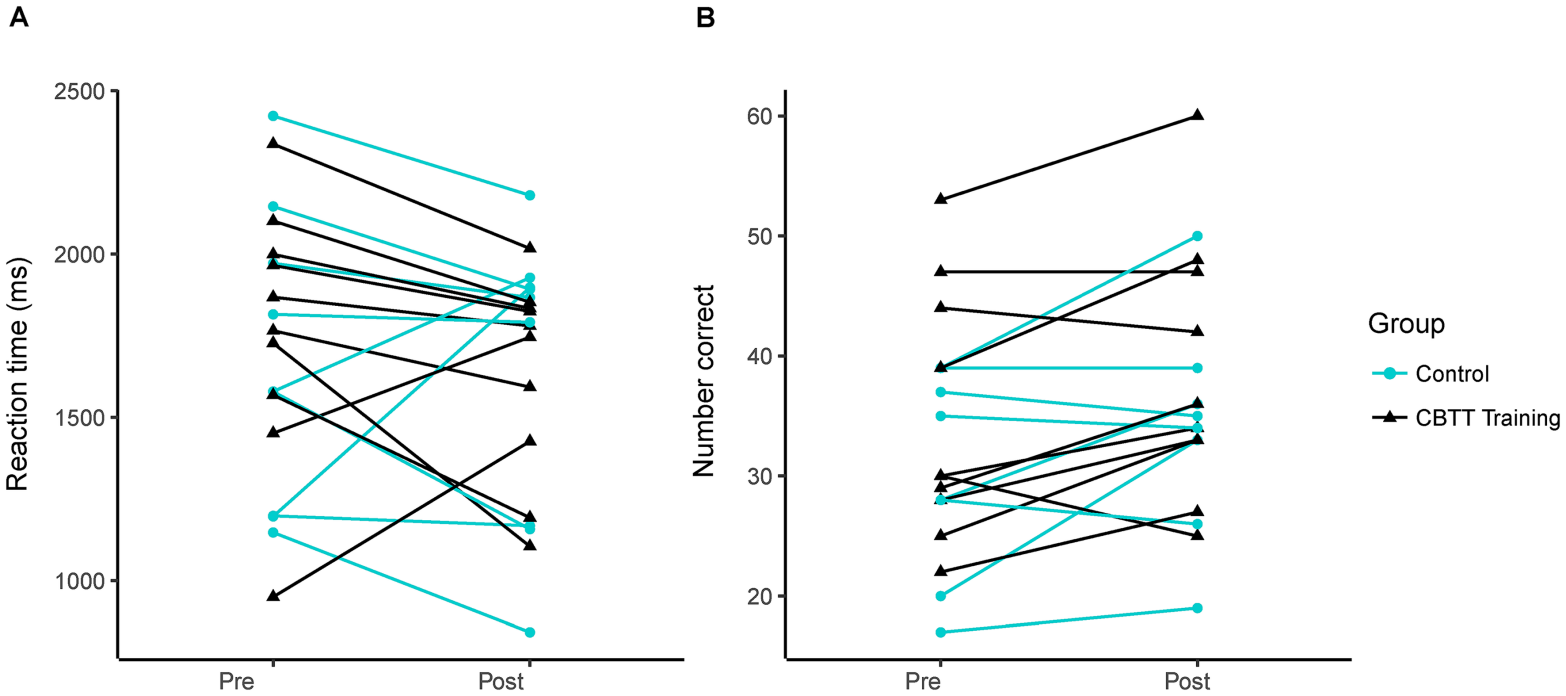
Mental rotation performance. Each line represents data from an individual participant. **(A)** Reaction time on pre- vs. post-test. **(B)** Number of correct trials on pre- vs. post-test.

The control group exhibited a slightly greater reduction in mental rotation reaction time compared to the experimental group at the post-test [Hedges’ g = 0.28 (−0.62, 1.19)]. However, we found no statistically significant between-group difference in mental rotation reaction time at the post-test [Figure 2A; time *β*(SE) = −56.45(107.5), *p* = 0.61; time by group interaction: *β*(SE) = −96.59(148.19), *p* = 0.52; control reaction time: 1637 ± 303 ms, training reaction time: 1636 ± 457 ms]. This suggests that twenty minutes of CBTT training did not impact mental rotation reaction time. Both groups had similar average starting reaction times [control: 1773 ± 387 ms, training: 1673 ± 453 ms; group *β*(SE) = 93.39(204.75), *p* = 0.65]. There was no effect of sex on performance [*β*(SE) = 40.01(103.03), *p* = 0.84].

Since mental rotation reaction time was not significantly different between groups, we investigated the potential effect of repeated mental rotation testing by calculating an effect size for paired samples. We found a small effect of repeated testing with an effect size of −0.26 [−0.75, 0.23]. However, because the 95% confidence interval crosses zero, there is likely a minimal effect of re-testing mental rotation after twenty minutes.

The training group showed a slightly greater change in the number of correct trials than the experimental group at the post-test [Hedges’ g = 0.25 (−0.66, 1.15)]. However, we found no statistically significant between-group difference in the number of correct trials [Figure 2B; group *β*(SE) = 3.63(5.20), *p* = 0.49; time *β*(SE) = 3.63(2.12), *p* = 0.11; time by group *β*(SE) = 0.18(2.84), *p* = 0.95; sex *β*(SE) = −1.57(5.10), *p* = 0.76]. This suggests that twenty minutes of CBTT training did not impact the number of correct trials on the mental rotation task. Mental rotation performance values for each participant can be found in Supplementary Table 1.

### 3.2 Lesion side did not impact CBTT performance

**Figure 3:**
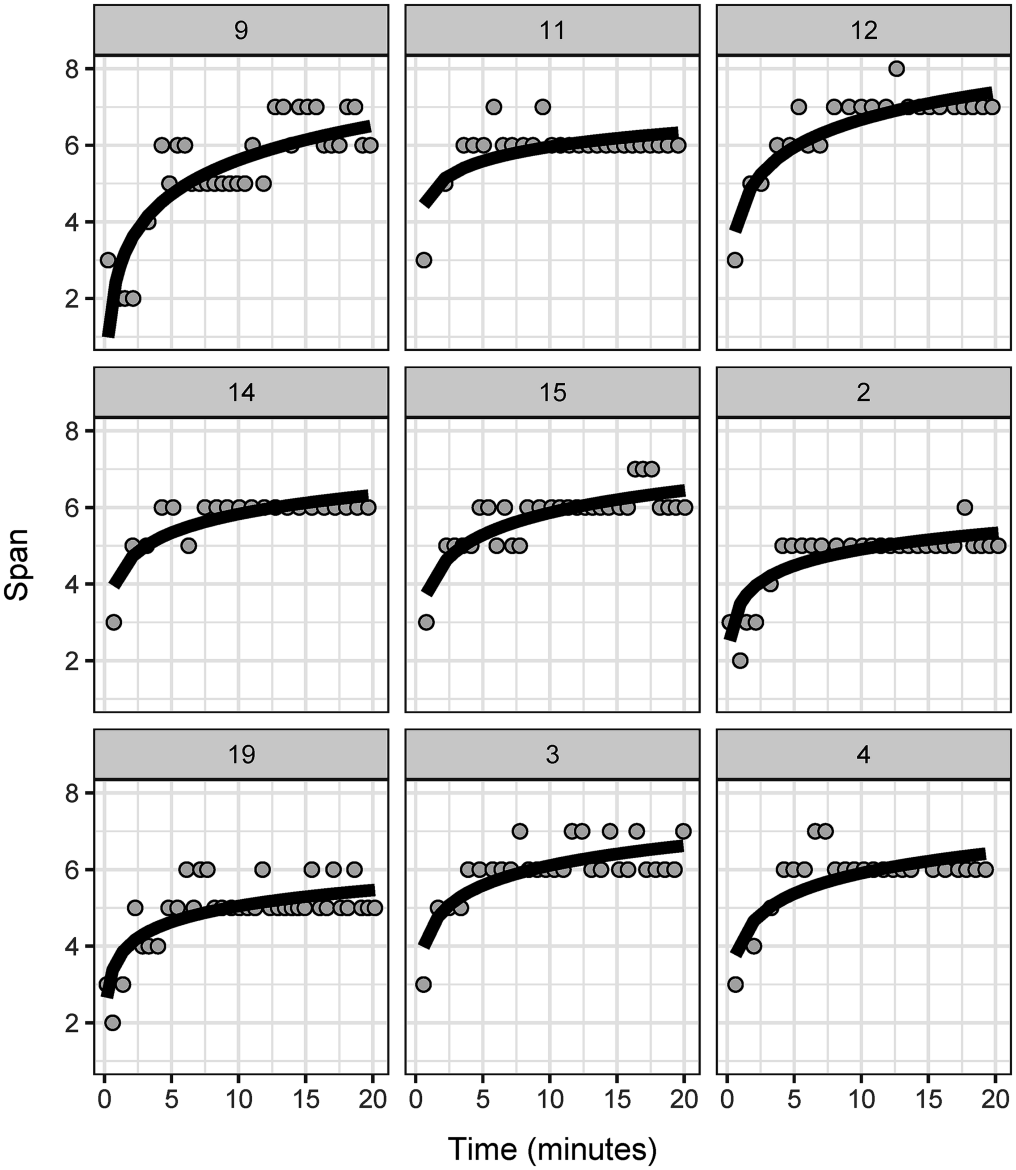
Corsi block tapping task performance across participants in the training group. Each panel shows Corsi block tapping task training data from an individual participant on correct trials. Individual models from the mixed effects model are plotted on each panel.

The maximum CBTT span ranged between six and eight, suggesting potential for further improvement with longer training periods. Similar to neurotypical adults (29), improvement in the CBTT over time in persons post-stroke followed a logarithmic trend (Figure 3; logarithmic BIC 486.2, linear BIC: 607.9). We found no effect of lesion side on CBTT performance [log(time) *β*(SE) = 0.78(0.16), *p* = 0.001; lesion side *β*(SE) = −0.38(0.52), *p* = 0.49; log(time) by lesion side *β*(SE) = 0.03(0.21), *p* = 0.89].

## 4 Discussion

We aimed to understand if a single, twenty-minute CBTT training session improved mental rotation performance in adults with stroke compared to a no-training control group of adults with stroke. We found that twenty minutes of CBTT training did not improve post-stroke mental rotation in this sample. This suggests that longer CBTT training bouts or a different type of visuospatial training may be necessary to improve mental rotation in adults with stroke.

Twenty minutes of CBTT training did not improve reaction time or number of correct trials in the mental rotation task. This contrasts with our previous work that found twenty minutes of CBTT training improved mental rotation performance in neurotypical young adults (29). Increased dosage of CBTT training may be necessary to improve mental rotation in adults post-stroke. There is evidence that individuals with cognitive impairment need a higher dosage of computerized cognitive training (45) than we provided in our study. For cognitively impaired older adults, the ideal dosage of a computerized cognitive training program that included CBTT (among other cognitive trainings) was between 45–50 min/day, 6 days/week (45). While the exact computerized cognitive training paradigm differed from the one used in our study, the results indicate that individuals with possible cognitive impairment (such as adults with stroke) may need higher dosage and frequency than younger neurotypical adults to see improvements in cognition. There is also previous work suggesting that listening to classical music can improve CBTT performance in neurotypical adults (46), suggesting that other interventions could be paired with CBTT training to potentially enhance visuospatial performance.

There was considerable variability in mental rotation performance within our sample, with some participants exhibiting significant reductions in reaction time at the post-test while others showed substantial increases in reaction time (Figure 2A). Individual variability and our relatively small sample size may have limited our ability to detect a clear group effect. Our work provides preliminary effect sizes for future research on mental rotation or CBTT training. Further research is needed to understand the factors that may contribute to between-individual variability.

It is also possible that a different visuospatial training paradigm may have a more substantial effect on mental rotation performance than only training with the CBTT. Though the CBTT and mental rotation both require visuospatial working memory, they may use different subsystems of visuospatial working memory (47, 48). Mental rotation primarily relies on the visual subsystem (48), where the CBTT primarily relies on the spatial subsystem (47). Thus, the transfer between tasks may be lower than a task that primarily trains the visual subsystem, such as a pattern span task (47). There is evidence that visuospatial working memory training (using a matrix task) has limited transfer to other visuospatial tasks (i.e., CBTT, Stroop test, etc.), particularly in neurotypical adults older than 75 years (49). It may be more beneficial to incorporate various visuospatial trainings to maximize improvements in visuospatial function after stroke.

We found that stroke lesion side did not impact CBTT performance. Previous work has found that individuals with right hemisphere lesions have worse CBTT performance compared to individuals with left hemisphere lesions (44). However, others have found no effect of lesion side (50, 51). This is in line with evidence that individuals with left hemisphere lesions can also have impaired spatial performance, specifically with spatial visualization (52, 53). Additionally, the CBTT is likely not a purely visuospatial task and may require executive function resources, particularly with longer sequence lengths (54). This suggests that brain areas outside of the right-dominant spatial areas of the brain may be active. Previous work has found that spatial memory was correlated with distributed bilateral damage to cortical and subcortical structures (51, 55). Spatial memory deficits also appear to be correlated with damage to functional networks and white matter tracts (51, 55, 56), which may contribute to why we did not find an effect of lesion side.

Our study had a few limitations. First, participants only completed a single, twenty-minute training session, limiting our ability to understand the impact of repeated CBTT training on mental rotation. Future work is needed to understand the optimal dosage and frequency of CBTT training. Second, our sample size was relatively small, with only nine participants completing the CBTT training. This limited our ability to rigorously assess factors that may impact CBTT performance, as smaller sample sizes may not accurately detect the experimental effect (57). However, this work provides data (see Supplementary Table 1) that can be used in future work to perform *a priori* power calculations to help determine an appropriate sample size (57).

In conclusion, we found that twenty minutes of CBTT training did not improve mental rotation in adults with stroke and that lesion side did not impact CBTT performance. More research is needed to understand the optimal dosage, frequency, and content for computerized cognitive training to improve visuospatial function in adults with stroke.

## Data Availability

The raw data supporting the conclusions of this article will be made available by the authors, without undue reservation.
