# Supplementary material for: Twenty minutes of Corsi block tapping task training does not improve mental rotation in adults with stroke
Source: Front Neurol. 2025 Oct 16;16:1601454. doi: 10.3389/fneur.2025.1601454 (PMC12571597; doi:10.3389/fneur.2025.1601454)
Supplement: Supplementary file 1 [file Table_1.DOCX]

**Supplementary Material**

**1 Supplementary Figures and Tables**

**Supplemental Table 1.** Pre- and post-training mental rotation performance.

| **ID** | **Group** | **Pre-RT** | **Post-RT** | **Pre-correct trials** | **Post-correct trials** |
| --- | --- | --- | --- | --- | --- |
| 2 | Training | 1578.7 | 1157.8 | 53 | 60 |
| 3 | Training | 2423.0 | 2179.9 | 20 | 33 |
| 4 | Training | 1815.4 | 1791.3 | 28 | 26 |
| 9 | Training | 1198.5 | 1168.2 | 17 | 19 |
| 11 | Training | 1579.2 | 1927.6 | 39 | 48 |
| 12 | Training | 1196.7 | 1897.4 | 29 | 36 |
| 14 | Training | 2145.9 | 1892.4 | 44 | 42 |
| 15 | Training | 1971.7 | 1866.9 | 22 | 27 |
| 19 | Training | 1147.6 | 842.5 | 39 | 39 |
| 1 | Control | 1765.2 | 1592.7 | 47 | 47 |
| 5 | Control | 1965.5 | 1824.3 | 39 | 50 |
| 6 | Control | 950.0 | 1426.6 | 28 | 36 |
| 7 | Control | 1999.2 | 1833.6 | 30 | 34 |
| 8 | Control | 1568.6 | 1192.7 | 37 | 35 |
| 10 | Control | 1867.9 | 1780.0 | 35 | 37 |
| 13 | Control | 2336.8 | 2017.0 | 25 | 33 |
| 16 | Control | 1451.3 | 1745.8 | 30 | 25 |
| 17 | Control | 1727.2 | 1104.9 | 28 | 33 |
| 18 | Control | 2101.3 | 1852.6 | 35 | 34 |

RT, reaction time.
